# Measurement of Lipid Oxidation in Foods and Selected Biological Systems Using LC-MS/MS-Based Lipidomics

**DOI:** 10.3390/antiox15101301

**Published:** 2026-10-08

**Authors:** Danyoung Kim, JuDong Yeo

**Affiliations:** Department of Food Science and Biotechnology of Animal Resources, Konkuk University, Seoul 05029, Republic of Korea; dan2002@konkuk.ac.kr

**Keywords:** lipid oxidation, LC-MS/MS, lipidomics, emulsion, oleosome, artificial intelligence

## Abstract

Lipid oxidation is an important factor affecting food quality and oxidative stability, but conventional oxidation indices provide limited information on individual oxidation products and changes in lipid species. This review focuses on liquid chromatography-tandem mass spectrometry (LC-MS/MS)-based approaches for lipid oxidation analysis and their applications in food and selected biological systems. LC-MS/MS provides molecular-level information on individual oxidation products and oxidized lipid species; however, its analytical reliability can be affected by hydroperoxide instability, matrix effects, limited availability of authentic standards, and variability in data processing and lipid annotation. The analytical characteristics of hydroperoxides, aldehydes, alcohols, ketones, organic acids, hydrocarbons, and oxidized complex lipids are described together with the advantages and limitations of LC-MS/MS analysis. In addition, this review compares targeted analysis with untargeted and pseudotargeted lipidomics and discusses their applications to edible oils, meat, emulsions, and oleosomes. The use of lipidomics in selected biological systems and artificial intelligence (AI)-assisted lipid analysis is also discussed. Therefore, combining validated targeted analysis and lipidomics with conventional oxidation indices may provide a more reliable and comprehensive evaluation of lipid oxidation.

## 1. Introduction

Lipids are major food constituents that determine the sensory properties and nutritional value of foods. However, during cooking, processing, and storage, lipids undergo oxidation induced by oxygen, heat, light, metal ions, and other factors, resulting in quality deterioration [1]. Lipid oxidation generally proceeds through initiation, propagation, and termination stages. During this process, hydroperoxides are first formed as primary oxidation products and are subsequently decomposed into various low-molecular-weight secondary oxidation products, including aldehydes, ketones, alcohols, organic acids and hydrocarbons [2,3,4].

Foods with a high content of polyunsaturated fatty acids (PUFAs) are particularly susceptible to oxidation. The resulting off-flavors and oxidation products, such as aldehydes and acrolein, can adversely affect not only the storage stability of foods but also consumer health [5]. Therefore, accurate assessment of the extent of lipid oxidation has long been considered an important indicator of food quality.

Conventional methods such as peroxide value (POV), thiobarbituric acid reactive substances (TBARS), p-anisidine value (p-AV), and conjugated diene value have been used to evaluate lipid oxidation. Although these methods are relatively simple and inexpensive, they generally provide only the total amount of specific functional groups, such as peroxides or aldehydes, and cannot distinguish among individual oxidation products. In addition, interference can occur when colored samples or complex food matrices are analyzed [6].

To overcome these limitations, liquid chromatography-tandem mass spectrometry (LC-MS/MS), including high-performance liquid chromatography-tandem mass spectrometry (HPLC-MS/MS) and ultra-high-performance liquid chromatography-tandem mass spectrometry (UHPLC-MS/MS), has increasingly been used to characterize lipid oxidation products [7,8]. LC-MS/MS is a general term describing the coupling of liquid chromatography with tandem mass spectrometry, whereas high-performance liquid chromatography (HPLC) and ultra-high-performance liquid chromatography (UHPLC) refer to chromatographic formats, with UHPLC generally using smaller particle sizes and higher operating pressures than conventional HPLC. The mass spectrometric platform should also be considered separately from the chromatographic format. Triple-quadrupole instruments are commonly used for selective targeted analysis using multiple reaction monitoring/selected reaction monitoring (MRM/SRM), whereas high-resolution mass spectrometry (HRMS) platforms such as quadrupole time-of-flight (QTOF) and Orbitrap-based instruments are widely used for accurate-mass analysis, structural annotation, and untargeted lipidomics. Accordingly, the analytical characteristics of LC-MS/MS depend on both the chromatographic and mass spectrometric configurations used. 

In practice, the choice of analytical strategy depends on the research question and the oxidation products of interest. Conventional oxidation indices are useful for rapid evaluation of overall oxidative status, whereas targeted LC-MS/MS is more suitable for selective analysis of defined oxidation products. Untargeted and pseudotargeted lipidomics provide broader molecular coverage. GC/GC-MS is mentioned only where relevant to volatile oxidation products. When both overall oxidation status and molecular-level information are required, a combined strategy can provide a more comprehensive evaluation. The major characteristics and appropriate applications of these analytical approaches are summarized in Table 1.

This review summarizes the analytical principles and significance of LC-MS/MS for lipid oxidation products and comprehensively discusses lipidomics-based approaches and their applications across food systems ranging from extensively studied matrices to more recently investigated food models. In addition, selected animal studies are discussed to extend the interpretation of lipid oxidation from food matrices to biological systems, where related oxidized lipid species are formed and analyzed under different matrix and metabolic conditions. The use of AI-assisted approaches for lipidomics data analysis is also discussed. The review therefore focuses on LC-MS/MS-based approaches, with GC-based methods mentioned only when necessary for selected volatile oxidation products.

To provide a more integrated interpretation of these studies, this review considers lipid oxidation through four connected levels of evidence: (i) the chemical formation and transformation of oxidation products, (ii) analyte stability and analytical detection, (iii) the structural evidence supporting lipid identification, and (iv) interpretation in food and biological systems. These levels are related but should not be treated as equivalent: a change in signal abundance does not necessarily establish the identity of an oxidation product, and structural identification alone does not demonstrate the mechanism by which it was formed.

## 2. Methods

This review was conducted as a narrative review using a structured literature search. Literature searches were performed using Google Scholar, with primary emphasis on peer-reviewed studies published from 2015 to September 2026. Search terms included combinations of “lipid oxidation,” “oxidized lipid,” “hydroperoxide,” “oxylipin,” “HPLC-MS/MS,” “LC-MS/MS,” “lipidomics,” “food,” “animal model,” and “machine learning.” Older landmark studies were also included when they established analytical principles or methods that remain in use. Studies were included or prioritized when they provided clear analytical conditions, validation data, mechanistic information, or applications directly relevant to lipid oxidation. Conference abstracts, non-English publications, studies mainly focused on unrelated contaminants, and studies lacking a clear analytical or mechanistic connection to lipid oxidation were excluded from the main discussion. The selected literature was organized into conventional oxidation indices, targeted LC-MS/MS analysis, untargeted and pseudotargeted lipidomics, and AI-assisted lipidomics and further classified according to oxidation-product class, sample matrix, and data-analysis workflow.

## 3. Analysis of Lipid Oxidation Using LC-MS/MS

### 3.1. Pre-Analytical Stability, Reactivity, and Method Validation

Pre-analytical handling and method validation are critical because sample preparation can alter the apparent oxidation profile, particularly for unstable hydroperoxides and reactive aldehydes. Exposure to heat, light, oxygen, and catalytic metals should be minimized where relevant, and extraction and storage should be performed at low temperature when compatible with the analyte and matrix. Internal standards should be added as early as practical, and stabilization or derivatization should be used when validated for the target analyte [8,9,10,11]. Quantitative methods should evaluate calibration performance, recovery, precision, LOD/LOQ, matrix effects, carryover, and analyte stability during extraction, storage, and autosampler residence. When substantial matrix effects are present, matrix-matched calibration or standard-addition approaches should be considered, with isotope-labelled internal standards preferred where available. These pre-analytical and validation factors should be reported together with the structural identification level so that differences among studies can be interpreted reliably.

Hydroperoxide decomposition and aldehyde reactivity are particularly important because they can change the apparent oxidation profile between sampling and instrumental analysis. Loss of hydroperoxides during extraction, storage, or autosampler residence can underestimate primary oxidation, whereas reactive aldehydes may be consumed through adduct formation with proteins, amino acids, or other nucleophilic matrix components. Conversely, artifactual oxidation during sample handling can increase measured oxidation products. For this reason, validation should be interpreted together with pre-analytical control: calibration and precision characterize instrumental performance, while recovery, matrix effects, analyte stability, carryover, and isotope-labelled internal standards determine whether the measured concentration adequately represents the original sample [8,9,10,11,12,13,14]. 

### 3.2. Hydroperoxides

**Figure 1:**
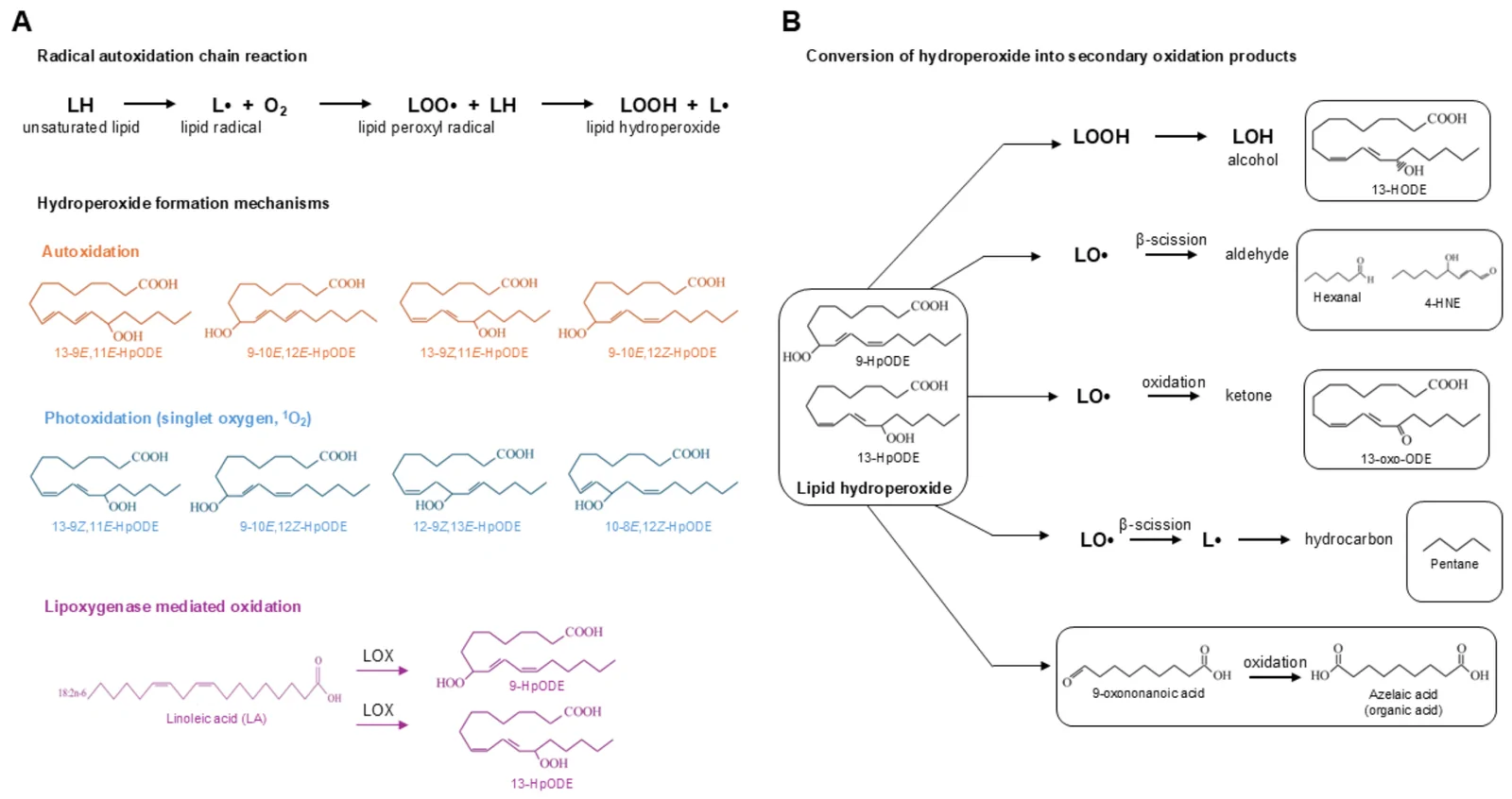
Formation of lipid hydroperoxides and representative secondary oxidation products. (**A**) Radical autoxidation chain reaction and representative hydroperoxide isomers formed through autoxidation, photooxidation, and lipoxygenase-mediated oxidation of linoleic acid. Orange, blue, and purple indicate autoxidation, photooxidation, and lipoxygenase-mediated oxidation, respectively. (**B**) Representative conversion pathways of lipid hydroperoxides to alcohols, aldehydes, ketones, hydrocarbons, and organic acids. Abbreviations: LOX, lipoxygenase; HpODE, hydroperoxyoctadecadienoic acid; HODE, hydroxyoctadecadienoic acid; 4-HNE, 4-hydroxy-2-nonenal; LOOH, lipid hydroperoxide; LO•, lipid alkoxyl radical.

Lipid hydroperoxides (LOOHs) are primary oxidation products formed when a radical abstracts a hydrogen atom from a carbon adjacent to a double bond in an unsaturated fatty acid, followed by the addition of oxygen. Depending on the method of hydroperoxide formation, lipid oxidation can be classified as autoxidation, photooxidation mediated by singlet oxygen, or enzymatic oxidation mediated by lipoxygenase (LOX) [7,15]. These hydroperoxide formation mechanisms and their subsequent conversion into representative secondary oxidation products are summarized in Figure 1**.** Even when derived from the same fatty acid, different hydroperoxide isomers are generated depending on the oxidation mechanism. For example, photooxidation of linoleic acid produces hydroperoxyoctadecadienoic acid (HpODE) isomers, including 13-(9Z,11E)-HpODE as well as 9-, 10-, and 12-HpODE, whereas autoxidation predominantly produces 13-(9Z,11E)-HpODE, 13-(9E,11E)-HpODE, 9-(10E,12Z)-HpODE, and 9-(10E,12E)-HpODE [16]. Therefore, analysis of hydroperoxide isomer composition can be used to trace the oxidation pathway that occurred in a food and thereby identify the cause of oxidation.

Hydroperoxides are not only early indicators of food rancidity but also precursors that subsequently decompose into secondary oxidation products such as aldehydes and ketones, thereby contributing to flavor deterioration and the formation of oxidation products with potentially adverse effects on human health [7,8,17]. Traditionally, hydroperoxide concentrations have been quantified using POV; however, this approach provides only an overall measure of lipid hydroperoxides and does not provide information on the oxidation mechanism [7]. More recently, electrospray ionization (ESI)-LC-MS/MS methods have been developed in which sodium ions are introduced in positive ESI mode to form [M + Na]^+^ adduct ions. Upon tandem mass spectrometry (MS/MS) fragmentation, bonds near the hydroperoxide group preferentially cleave to generate distinct fragment ions, enabling discrimination among isomers such as 9-, 10-, 12-, and 13-HpODE [7]. In addition, differential-energy qualifier-ion monitoring by HPLC-ESI-MS/MS, in which multiple collision energies are applied during MS/MS analysis, has been established to distinguish and quantify hydroperoxides, hydroxides, and ketones based on their characteristic fragment ions [18]. Furthermore, an LC-MS/MS method using a C4 reversed-phase column and selected reaction monitoring has been used for the separation and quantification of fatty acid hydroperoxides (FAOOHs) at low picomolar levels [8]. LC-MS/MS-based hydroperoxide analysis provides important information for elucidating lipid oxidation mechanisms in foods and developing strategies to prevent oxidation [19]. However, hydroperoxides can decompose during extraction and extended autosampler residence [8,12], while antioxidant addition during sample handling may limit oxidation [20]. In the LC-MS/MS method for quantitative determination of FAOOHs described by Liang et al. [8], calibration showed R^2^ values of 0.965–0.998, with limits of detection (LODs) of 0.1–1 pmol/µL, limits of quantification (LOQs) of 1–2.5 pmol/µL, recoveries of 48.1–76.0%, and intra- and inter-day coefficients of variation (CVs) below 15% [8]. Matrix effects differed among FAOOH species, including both ion suppression and enhancement [8]. FAOOHs were derivatized with 2-methoxypropene for 10 min at room temperature before alkaline hydrolysis to improve the stability of the hydroperoxide group during sample processing, and fatty acid (FA) 19:1-OOH was added as an internal standard before sample processing [8]. FAOOH standards were also reported to be unstable during extended storage even at −30 °C, and prolonged autosampler residence increased the possibility of degradation [8]. Therefore, reliable hydroperoxide quantification requires appropriate calibration and internal-standard use together with evaluation of recovery, matrix effects, precision, and analyte stability during extraction, storage, and LC-MS analysis. 

### 3.3. Aldehydes

Aldehydes are major secondary oxidation products generated when hydroperoxides form alkoxyl radicals and subsequently undergo β-scission, as illustrated in Figure 1B, and include malondialdehyde (MDA), 4-hydroxy-2-nonenal (4-HNE), 4-hydroxy-2-hexenal (4-HHE), acrolein, and hexanal. Among these, MDA and 4-HNE are highly reactive toward amino acid residues such as lysine and histidine and can induce protein adduct formation and cross-linking. These changes can alter protein structure and function and affect food quality [13]. Indeed, an LC-ESI-MS/MS study of soybean-derived peptides confirmed the formation of adducts through the binding of MDA and 4-HNE to amino acid residues [21].

TBARS has been widely used for aldehyde analysis; however, the assay has low selectivity for MDA and may also react with sugars, amino acids, and other carbonyl compounds, which can compromise quantitative accuracy [22,23]. Therefore, TBARS should not be interpreted as specific MDA quantification without chromatographic confirmation. Accordingly, LC-MS/MS methods for aldehyde quantification have been developed. For example, aldehydes in soybean oil and fried foods were detected and quantified by liquid chromatography-mass spectrometry (LC-MS) following derivatization with 2-hydrazinoquinoline [9].

These aldehydes are useful not only for evaluating the degree of food oxidation but also for elucidating interactions between proteins and lipids during oxidation [13]. However, highly reactive aldehydes may be lost during sample preparation or react with food constituents, resulting in underestimation [14]. In an LC-MS method for aldehydes in frying oils and fried foods, aldehydes were derivatized with 2-hydrazinoquinoline at 60 °C for 30 min, and acetone-d_6_ was used as a deuterated isotope-labelled internal standard for quantification [9]. In another method, 35 aldehydes and ketones in fish oil were analyzed after 2,4-dinitrophenylhydrazine (2,4-DNPH) derivatization and solid-phase extraction (SPE) cleanup [24]. Matrix-specific validation is also important; HPLC-MS/MS analysis of 4-HNE in pork products showed a linear range of 0.1–10 mg/kg, an LOD of 0.043 mg/kg, recovery of approximately 60%, and intra- and inter-day precision below 10% relative standard deviation (RSD) [10]. Therefore, appropriate derivatization, isotope-labelled internal standards, and matrix-specific recovery evaluation are important for reliable aldehyde quantification.

### 3.4. Alcohols

Hydroxy fatty acids (LOHs), which are products of lipid oxidation, are formed when hydroperoxides undergo homolysis to generate alkoxyl radicals that subsequently acquire hydrogen and are reduced [25], or when the hydroperoxides themselves are reduced [12,26]. Some of the resulting hydroxy fatty acids also contribute to the formation of off-flavors in foods [27,28,29].

GC-MS has also been used after derivatization [30,31]; however, LC-MS enables hydroxy fatty acids to be analyzed in intact lipid molecules such as triacylglycerols (TGs) without derivatization [32], thereby providing information on the glycerol backbone [2]. Atmospheric pressure chemical ionization (APCI) and ESI are commonly used as MS ionization techniques [2,33]. In particular, during APCI-MS analysis, fatty acyl bonds at specific sn positions of the TG glycerol backbone can undergo preferential dissociation, generating sn-position-dependent fragment ion patterns [33,34]. Measurement of the intensity ratios of these fragment ions can provide quantitative information on the positional distribution of fatty acids on the glycerol backbone [34].

As downstream lipid oxidation products, alcohols can provide additional information on oxidation pathways that cannot be obtained from hydroperoxides or aldehydes alone. The positional patterns of hydroxy fatty acids can also serve as oxidation-pathway markers because different oxidation mechanisms can produce characteristic positional isomers [11]. However, because many hydroxy fatty acids occur at low concentrations, sensitive detection and separation of regio- and stereoisomers are required [11,35]. In a validated LC-MS/MS method for hydroperoxydocosahexaenoic acid (HpDoHE) and hydroxydocosahexaenoic acid (HDoHE) isomers, 5(S)-hydroxyeicosatetraenoic acid-d8 [5(S)-HETE-d8] and 12(S)-hydroxyeicosatetraenoic acid-d8 [12(S)-HETE-d8] were used as isotope-labelled internal standards, and calibration was performed using analyte-to-internal-standard peak area ratios [11]. HDoHEs showed LODs of 0.5–8.5 pg and r^2^ values of 0.99 or greater, with average recoveries of 84 ± 20% in brain and 90 ± 13% in plasma [11]. In comparison, HpDoHEs showed lower recoveries of 16 ± 22% and 22 ± 14%, respectively, indicating differences in analyte stability and matrix effects [11]. Most tested isomers also showed RSD values below 8% and accuracy above 90% [11]. Therefore, reliable analysis of hydroxy fatty acids requires appropriate internal-standard correction, isomer-specific separation, and evaluation of recovery and matrix effects.

### 3.5. Ketones, Epoxy- and Oxo-Lipids, and Organic Acids

Ketones are secondary oxidation products generated during hydroperoxide decomposition. Oxidation or rearrangement of alkoxyl-radical intermediates can generate carbonyl-containing products, including ketones and oxo-fatty acids [25,36]. Epoxy fatty acids are also formed during the oxidation of unsaturated lipids and represent another class of oxygenated lipid oxidation products [2]. Both epoxy- and oxo-fatty acids can provide additional information on the oxidation of unsaturated fatty-acyl chains. Organic acids are formed during advanced stages of lipid oxidation when secondary products undergo further oxidation [36,37]. These products can contribute to rancid and other off-odors and thereby affect the sensory quality of foods [29].

Although GC-MS has been used for derivatized ketones [38,39], LC-MS methods have been applied to ketones and organic acids either directly or with analyte-specific derivatization [18,40]. LC-MS-based methods can also be used for the analysis of epoxy- and oxo-lipids, providing molecular information on these oxygenated lipid oxidation products [2]. C18 reversed-phase chromatography is commonly used for less-polar analytes, whereas hydrophilic interaction liquid chromatography (HILIC) or other polar separations can improve the retention of short-chain organic acids [41,42]. Negative-mode ESI is frequently used for deprotonated organic acids [43].

These oxidation products are particularly useful for investigating relationships with sensory quality, including rancid and other off-odors [44]. However, ketones and organic acids span wide ranges of polarity and molecular weight, making it difficult to analyze all compounds simultaneously using a single chromatographic condition. Consequently, untargeted analysis can provide broader coverage of diverse oxidation products, including epoxy- and oxo-lipids [45,46]. For example, a validated LC-MS method for six organic acids showed R^2^ > 0.991, an LOQ of 0.01 mM, CVs of 2–11% in standards, and recoveries of 95–105% in fermentation medium [43]. The study also recommended analysis within 24 h after extraction because longer storage increased analytical variability [43]. 

### 3.6. Hydrocarbons

Hydrocarbons are secondary oxidation products generated from the carbon chain opposite the aldehyde-forming fragment when alkoxy radicals undergo β-scission during lipid oxidation [47]. Representative hydrocarbons include pentane and ethane. Pentane is known to form during the decomposition of hydroperoxides derived from PUFAs such as linoleic acid [48], whereas ethane is produced mainly during the degradation of n-3 fatty acids. Thus, the type of hydrocarbon formed during oxidation can provide information on the fatty acid from which oxidation originated [48,49].

Hydrocarbons are relatively unreactive and stable and are not readily metabolized in vivo; therefore, some studies have proposed them as more reliable in vivo indicators than aldehydes such as hexanal, which are more readily metabolized [50]. However, low-molecular-weight hydrocarbons are highly nonpolar and volatile and lack readily ionizable functional groups, making them difficult for LC-MS/MS analysis [51]. For this reason, headspace GC/GC-MS is briefly considered as an alternative for volatile hydrocarbons such as pentane and ethane [52].

Polycyclic aromatic hydrocarbons (PAHs) are not direct lipid oxidation products formed through β-scission of lipid hydroperoxides and are therefore not discussed further in this review [53,54].

## 4. Application of Lipidomics to Lipid Oxidation Analysis

### 4.1. Introduction to Lipidomics

Lipidomics is an omics approach based on mass spectrometry (MS) that characterizes the composition and abundance of lipids within a sample to evaluate global changes in the lipidome [55]. Depending on the analytical objective and data-acquisition strategy, lipidomics can be performed using targeted, untargeted, or pseudotargeted approaches, as discussed in Section 4.2. Lipidomics is a branch of metabolomics that focuses on the comprehensive analysis of lipid species and their roles as structural components of cell membranes, energy storage molecules, and signaling mediators [56]. Lipid species are generally classified into eight major categories as defined by the LIPID MAPS system: fatty acyls, glycerolipids (GLs), glycerophospholipids, sphingolipids, sterol lipids, prenol lipids, saccharolipids, and polyketides [56]. Recent advances in soft ionization techniques such as ESI and APCI, together with high-resolution mass spectrometry (HRMS) and tandem MS, have enabled the broad detection of lipid classes with diverse polarities and their individual molecular species, including TGs, phospholipids (PLs), sphingolipids, and free fatty acids [6].

Food lipids are primarily composed of neutral lipids, mainly TGs, polar lipids such as PLs, and various minor lipid components. Each lipid class comprises a diverse range of molecular species, largely determined by variations in the chain length and degree of unsaturation of their constituent fatty acids [57]. Lipid molecules undergo substantial changes during food processing steps such as fermentation, roasting, steaming, and boiling [58]. MS-based lipidomics has therefore been widely applied to detect food adulteration, monitor quality control, and screen processing-induced structural changes in lipid species [58]. In particular, during food processing and storage, oxidation can cause decreases in PUFA-containing TGs while simultaneously generating diverse oxidized lipids, including hydroperoxy-, hydroxy-, epoxy-, and oxo-lipids. Oxidation can also occur while fatty-acyl chains remain esterified within complex lipids, resulting in oxidized phospholipids (OxPLs) and oxidized triacylglycerols (OxTGs). Unlike free secondary oxidation products, OxPLs and OxTGs retain information on both the original lipid class and the oxidized fatty-acyl chain. LC-MS/MS-based lipidomics can therefore provide molecular-level information on changes in intact oxidized lipid species as well as their corresponding native lipids. This is particularly useful for evaluating lipid oxidation in complex food and biological matrices. Therefore, simultaneous analysis of native lipids and their oxidation derivatives is important for understanding lipid oxidation processes [59].

LC-MS/MS-based lipidomics reduces interference from complex lipid mixtures through chromatographic separation and provides retention time and MS/MS fragmentation information, allowing higher-confidence lipid identification [55]. This chromatography-coupled strategy also resolves different lipid classes and their molecular species within a single injection [58]. Such approaches have identified over a hundred individual lipid species in complex biological or food matrices in a single analysis [59]. However, lipid identification can remain uncertain when authentic standards are unavailable, and analytical results can also be affected by ion suppression, batch effects, and database- or software-dependent annotation. These limitations should therefore be considered when interpreting lipidomics data. Consequently, LC-MS/MS-based lipidomics is widely used to investigate changes in oxidized lipids in complex matrices such as foods. This approach has also been applied to animal models to characterize diet- or treatment-induced changes in tissue and serum lipid profiles [60]. As lipidomic datasets have grown in volume and complexity, machine learning and other AI-based approaches have increasingly been used to identify lipid patterns and biomarkers [61].

### 4.2. Advantages of Lipidomics

Targeted and untargeted approaches have different analytical objectives. Targeted LC-MS/MS focuses on predefined oxidation products, such as HpODE isomers, TGOOHs, PCOOHs, 4-HNE, and oxylipins, and is suitable for quantitative analysis when compound-specific standards, calibration, and validation are available. In contrast, untargeted lipidomics provides broader profiling of native and oxidized lipid species, generally using HRMS, and is mainly applied to the discovery of lipid changes associated with oxidation. Pseudotargeted lipidomics provides an intermediate strategy in which reproducible features identified by untargeted analysis are converted into a predefined multianalyte panel [62]. Therefore, these approaches are complementary and should be selected according to the analytical objective. Another advantage of lipidomics is that lipid species data can be integrated with multivariate statistical analysis. Principal component analysis (PCA) can be used to visualize changes in the overall lipid profile according to storage time or treatment conditions, whereas partial least squares discriminant analysis (PLS-DA) and orthogonal projections to latent structures discriminant analysis (OPLS-DA) can identify lipid species that contribute strongly to discrimination among treatment groups [63,64,65]. For instance, lipidomic profiling of walnut oil during accelerated storage revealed significant decreases in TGs, diacylglycerols (DGs), and phosphatidylcholines (PCs), together with changes in other lipid classes, characterizing the progression of oxidative rancidity over time [66]. When variable importance in projection (VIP) score, statistical significance testing, and correlation analysis are additionally applied, biomarkers closely associated with storage duration or the degree of oxidation can be selected [65]. Thus, lipidomics can be used not only to characterize changes in lipid composition but also to identify oxidation markers indicative of oxidative status.

Identified lipid species can be linked to databases such as the Kyoto Encyclopedia of Genes and Genomes (KEGG) and LIPID MAPS to determine which lipid metabolic pathways, including glycerolipid metabolism, glycerophospholipid metabolism, and fatty acid metabolism, are predominantly affected [67]. In the same walnut oil storage study, KEGG pathway analysis identified glycerophospholipid metabolism as a key pathway associated with oxidative rancidity [66]. In addition, correlation or network analyses can be used to evaluate relationships between lipid species and their associated oxidation or flavor products, thereby facilitating the identification of potential lipid precursors [68,69]. In frying oils, correlation analyses in thermally processed vegetable oils showed that oxidation was more pronounced in unsaturated TGs with abundant free radicals and low short-chain fatty acid content [70].

**Figure 2:**
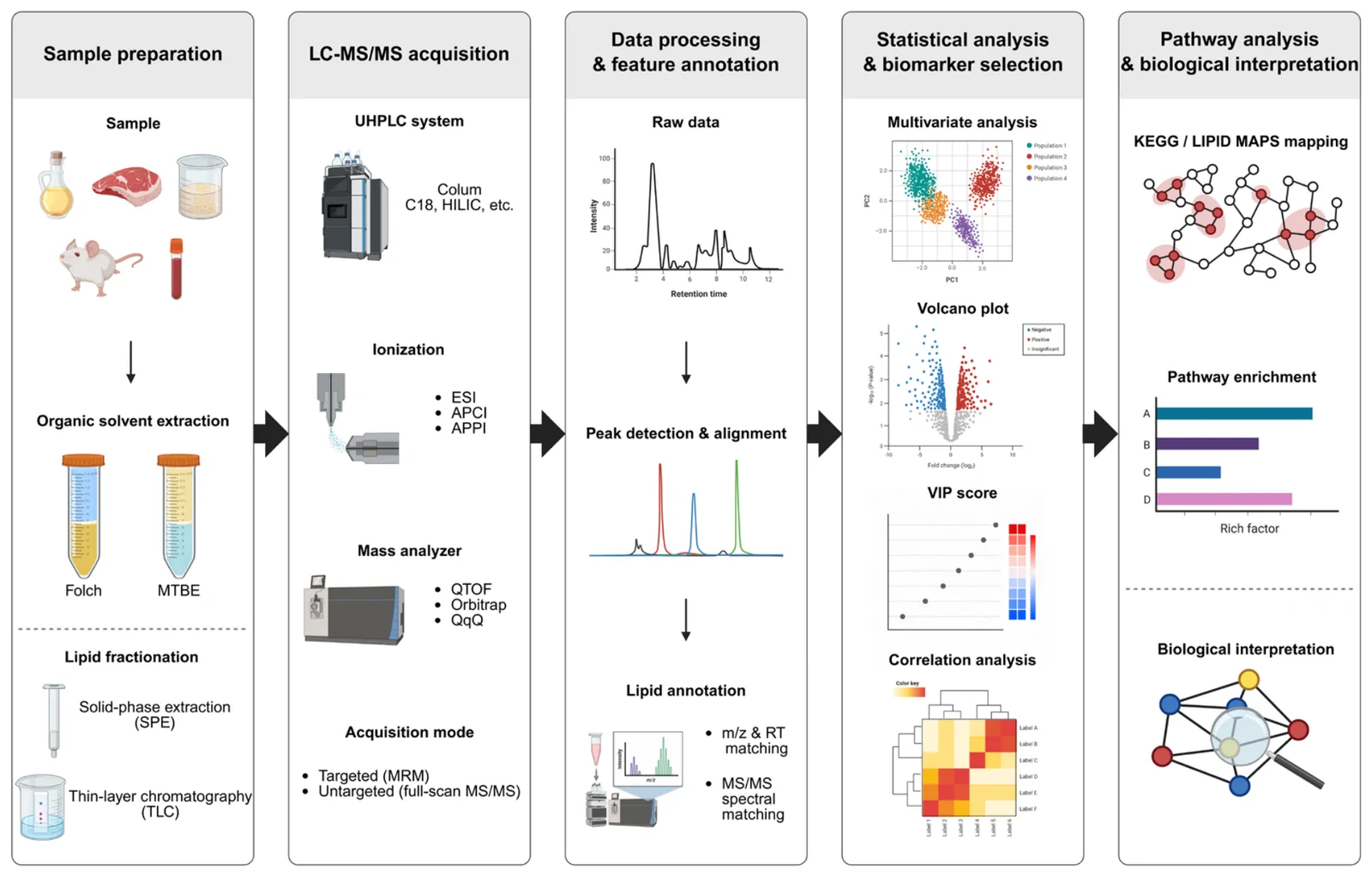
LC-MS/MS-based lipidomics workflow for lipid oxidation analysis. The workflow includes sample preparation and lipid extraction/fractionation, LC-MS/MS acquisition, peak detection and alignment, lipid annotation using m/z, retention time (RT), MS/MS spectra, statistical analysis and biomarker selection, and pathway/network-based biological interpretation. Abbreviations: ESI, electrospray ionization; APCI, atmospheric pressure chemical ionization; APPI, atmospheric pressure photoionization; QTOF, quadrupole time-of-flight; MRM, multiple reaction monitoring; SPE, solid-phase extraction; TLC, thin-layer chromatography; MTBE, methyl tert-butyl ether; KEGG, Kyoto Encyclopedia of Genes and Genomes.

However, pathway databases are constructed mainly around enzyme-catalyzed reactions in living systems and therefore cannot be assumed to represent the non-enzymatic radical chemistry occurring during food oxidation [71,72]. A KEGG enrichment result such as “glycerophospholipid metabolism” indicates that lipids assigned to that biological pathway changed; it does not by itself demonstrate the chemical sequence by which a hydroperoxide, aldehyde, OxTG, or OxPL was formed. Chemical interpretation should instead consider fatty-acyl unsaturation, known hydroperoxide decomposition routes, oxygen-addition patterns, isomer distributions, and correlations between precursor and product species. Biological pathway enrichment and chemical oxidation-pathway interpretation should therefore be reported as distinct layers of evidence. For example, increases in lysophospholipids may reflect phospholipid hydrolysis or lipid remodeling rather than direct lipid oxidation, whereas HETE species may arise through both enzyme-mediated lipid metabolism and non-enzymatic oxidation. Accordingly, changes in LPC, HETE, or other oxidation-associated lipids should not be interpreted as evidence of a specific oxidation or metabolic pathway without additional support from product isomers, precursor–product relationships, enzyme-related information, and complementary oxidation measurements. A general LC-MS/MS-based lipidomics workflow for lipid oxidation analysis is shown in Figure 2.

### 4.3. Identification Confidence and Annotation Levels

Oxidized-lipid identification requires careful interpretation because positional, geometrical, and stereochemical isomers can have the same elemental composition and similar MS/MS spectra. Accurate mass alone is therefore insufficient for complete structural identification. Retention time and diagnostic MS/MS fragment ions can increase identification confidence, whereas assignment of fatty-acyl composition, sn-position, double-bond position, or oxidation position requires corresponding structural evidence [55]. Therefore, oxidized lipids should be annotated only at the structural level supported by the analytical data. When specific structural features cannot be resolved, the lipid should not be reported as a fully identified molecular species. Comparison with authentic standards and additional chromatographic or MS/MS evidence can further improve identification confidence.

### 4.4. Oxidized Phospholipids and Oxidized Triacylglycerols

Oxidized phospholipids (OxPLs) and oxidized triacylglycerols (OxTGs) are intact complex lipids in which oxidation occurs while the susceptible fatty-acyl chain remains esterified to the phospholipid or glycerol backbone. Unlike free fatty acid oxidation products or low-molecular-weight secondary products, these species retain information on both the parent lipid class and the oxidized fatty-acyl moiety. OxPLs are particularly relevant to membrane- and interface-rich systems, whereas OxTGs are important in neutral-lipid-rich matrices such as edible oils and lipid droplets [17,19,59].

For food systems, TGOOH and PCOOH isomer analysis has demonstrated that intact oxidized lipids can provide mechanistic information that is not available from global indices alone. TGOOH isomer distributions have been used to distinguish radical- and singlet-oxygen-mediated oxidation in edible oils and milk, whereas PCOOH isomers have been used to examine phospholipid oxidation in mayonnaise [19,73,74,75]. Untargeted oxidative lipidomics can extend this coverage to broader OxTG and OxPL profiles, although confident assignment becomes more difficult as the number of possible oxidation sites and isomers increases [59,76,77].

In biological systems, OxPLs and other oxidized complex lipids can arise in membranes and lipoproteins under enzymatic and non-enzymatic oxidative conditions and may also participate in signaling or disease-associated processes [17,78,79,80,81]. Accordingly, an oxidized complex lipid detected by LC-MS/MS should not be interpreted solely from accurate mass. Retention behavior, diagnostic MS/MS fragments, oxidation-site evidence, and, when available, authentic standards are needed to support the reported structural level. Quantification is additionally limited by the scarcity of compound-specific standards and by class- and species-dependent ionization and matrix effects. These issues make OxPLs and OxTGs particularly important examples of why oxidation chemistry, identification confidence, and biological interpretation should be evaluated together.

### 4.5. Scope and Applicability of Lipidomics in Lipid-Containing Foods

In food research, lipidomics is used to evaluate lipid stability and oxidation characteristics by identifying changes in individual lipid species during processing and storage [82]. Since food lipid composition varies markedly according to raw material and processing conditions, it is important to determine which lipid species containing specific fatty acids selectively increase or decrease within the same lipid class [63]. Accordingly, studies commonly compare the relative abundances of TG, DG, PC, phosphatidylethanolamine (PE), lysophosphatidylcholine (LPC), and other lipid classes before and after treatment or over storage periods [83,84,85].

In lipid stability studies, changes can be evaluated by considering not only the abundance of lipid classes but also the fatty acyl composition of individual lipid species [86]. TGs and PLs containing PUFAs can decrease selectively during storage and processing, and the magnitude of these changes differs according to the degree of unsaturation [87,88].

Time-dependent changes in lipid profiles are also important in oxidation analysis. A study examining lipidome changes during refrigerated storage found that GL and PL compositions changed continuously with storage duration, allowing different stages of quality deterioration to be distinguished [83].

Lipidomics has also been applied to characterize lipid changes caused by food processing methods. In spray-dried egg yolk, quantitative LC-MS/MS-based lipidomics was used to compare individual lipid species before and after processing, revealing changes in specific PLs and GLs [89]. In thermally processed foods, lipid profiles before and after heating have also been compared to distinguish heat-sensitive from relatively stable lipid species and interpret these changes from the perspective of lipid oxidation [90]. Such studies demonstrate that lipid stability can be evaluated by applying different processing conditions to the same raw material. However, the oxidation products detected and their analytical relevance can differ depending on lipid composition and physical organization of the food matrix. Therefore, LC-MS/MS-based lipidomics should be interpreted according to matrix-specific characteristics rather than using the same analytical targets across different food systems. The major lipid characteristics, lipidomic changes, and applications in different food matrices are summarized in Table 2.

## 5. Lipid Oxidation Analysis in Diverse Food Models

The rate and mechanism of oxidation vary considerably depending on the physicochemical structure of a food, including whether lipids are surrounded by membranes such as PL layers and whether they are present in an emulsion [91]. Accordingly, LC-MS/MS-based analytical approaches have been widely applied not only to edible oils but also to meat, emulsions, and, more recently, oleosomes. Edible oils and meat were selected as well-established food models that have been widely studied in lipid oxidation research. Emulsions were included as representative interfacial lipid systems, whereas oleosomes were selected as naturally structured lipid droplets that have received increasing research attention. These models were therefore selected to represent different lipid organizations and research applications rather than all lipid-rich foods. The following sections describe how LC-MS/MS-based lipid oxidation analyses have been applied to these different food models.

### 5.1. Edible Oils

Edible oils are composed predominantly of TGs, and oxidation generates triacylglycerol hydroperoxides (TGOOHs), in which hydroperoxide groups are formed on fatty acid chains esterified to TGs, as major primary oxidation products [19,20]. Compared with other food models, edible oils contain relatively little matrix interference from proteins or carbohydrates, and oxidized lipids in these systems have therefore been extensively analyzed by LC-MS/MS.

In targeted analyses, linoleic acid hydroperoxide (HpODE) isomers in soybean, rice bran, and olive oils were separated by chiral LC-MS/MS, demonstrating that different oxidation mechanisms may predominate among edible oils [7]. TGOOH isomers in canola oil were also directly analyzed by LC-MS/MS to evaluate radical and singlet oxygen-mediated mechanisms involved in TG oxidation [19]. This approach was subsequently applied to extra-virgin olive oil, where differences in TGOOH isomer ratios generated by thermal and photooxidation were identified [73]. In addition, 35 aldehydes and ketones in commercial fish oil products were simultaneously quantified by LC-MS/MS after 2,4-dinitrophenylhydrazine (2,4-DNPH) derivatization and solid-phase extraction (SPE) cleanup, providing information on the oxidative stability of the products [24].

Lipidomics has also been used to investigate global changes in lipid composition during oxidation of edible oils. In flaxseed oil, changes in GLs, PLs, and other lipid species before and after roasting were analyzed using ultra-performance liquid chromatography (UPLC)-Q Exactive Orbitrap MS-based lipidomics [91]. Similarly, UPLC-QTOF-MS-based lipidomics was applied to hazelnut oil during accelerated storage to characterize changes in TG, DG, PC, and PE species [92]. Compared with untargeted lipidomics, direct analysis of HpODE or TGOOH isomers provides more specific information on oxidation pathways but is limited to predefined compounds and requires appropriate standards [7,19,73]. In contrast, untargeted lipidomics provides broader coverage of changes in native and oxidized lipid species, although lipid annotation and quantitative interpretation can be more limited [6,91,92]. Therefore, these approaches provide complementary information on specific oxidation products and broader lipidome changes.

### 5.2. Meats

Meat contains PLs and TGs in muscle and adipose tissues together with strong pro-oxidant factors, particularly heme iron from myoglobin [44]. Heme iron can promote lipid oxidation, particularly in PUFA-rich membrane PLs. In particular, aldehydes generated during oxidation can react with amino acid residues in muscle proteins, altering protein structure and function and thereby contributing to quality deterioration [13,44]. Thus, lipid and protein oxidation can occur simultaneously in muscle foods, and reactive lipid-derived aldehydes may also be associated with protein oxidation [13].

Sample preparation in meat also requires consideration of the complex muscle matrix. For example, methods developed for 4-HNE analysis in biological fluids showed unsatisfactory recovery and repeatability when applied to meat samples, and a meat-specific method using SPE cleanup and HPLC-MS/MS was therefore developed [10]. In targeted analysis, 4-HNE was quantified in pork and processed pork products using this method, and product-dependent differences in 4-HNE formation were observed [10]. The method showed an LOD of 0.043 mg/kg, approximately 60% recovery, linearity over 0.1–10 mg/kg, and intra- and inter-day precision below 10% relative standard deviation (RSD) [10], demonstrating the importance of matrix-specific sample preparation and validation in meat analysis.

More recently, lipidomics has increasingly been used to characterize broad changes in lipid classes in meat. In yellow-feathered chicken, UPLC-ESI-MS-based lipidomics was used to investigate changes in TG, DG, PL, and other lipid species during thermal processing. The study reported decreases in some unsaturated PLs and increases in lysophospholipids, which were attributed primarily to PL hydrolysis [93]. These changes should therefore be interpreted with caution, as they may reflect processing-related lipid remodeling rather than direct evidence of lipid oxidation. In precooked Chinese stewed beef, UPLC-ESI-MS/MS-based lipidomics was used to identify lipid precursors associated with warmed-over flavor (WOF) and demonstrate relationships between their changes, lipid oxidation, and off-flavor formation [94]. Thus, in meat systems, targeted analysis can quantify changes in specific oxidation products, whereas lipidomics can simultaneously characterize lipid precursors involved in oxidation and broader alterations in the lipidome.

### 5.3. Emulsions

Emulsions are food systems in which oil and water are stabilized by emulsifiers and are classified according to the continuous and dispersed phases as oil-in-water (O/W), in which oil is dispersed in water, or water-in-oil (W/O), in which water is dispersed in oil. O/W emulsions have a large oil-water interfacial area, facilitating interactions between lipids and metal ions or oxidation catalysts present in the aqueous phase; consequently, their oxidation behavior can differ from that of bulk oils [95]. Lipid oxidation in O/W emulsions occurs mainly at or near the oil-water interface, where unsaturated lipids can interact with transition metals and other pro-oxidants in the aqueous phase. The type and properties of the emulsifier can influence interfacial charge and metal binding, thereby affecting metal-catalyzed lipid oxidation [95]. Therefore, emulsions containing the same oil can show different oxidation behavior depending on their interfacial composition.

In canola oil-based infant formula and infant formula fortified with docosahexaenoic acid (DHA) ethyl ester, UPLC-MS/MS-based targeted analysis of free oxylipins revealed differences in oxidized fatty acid formation depending on DHA supplementation [96]. In a subsequent refrigerated storage study, both free and esterified oxylipins were quantified, showing that oxylipin levels changed with storage time and that DHA supplementation influenced the formation of certain oxidation products [97].

In mayonnaise, total lipids were extracted, PLs were fractionated using aminopropyl SPE, and the resulting fraction was analyzed by HPLC-MS/MS [74]. Analysis of phosphatidylcholine hydroperoxide (PCOOH) isomer composition suggested that PL oxidation in mayonnaise was initiated predominantly by radical oxidation rather than by singlet oxygen [74]. In milk, LC-MS/MS quantification of TGOOH isomers has likewise been used to elucidate oxidation pathways of milk fat [75].

Cheese is a complex protein gel matrix containing dispersed fat globules derived from the original milk emulsion. In irradiated Camembert cheese, UHPLC-Q-Orbitrap-MS-based lipidomics was used to characterize oxidized lipids including oxidized triacylglycerol (OxTG) and oxidized diacylglycerol (OxDG) species, and identify lipid species that changed in response to irradiation [76]. Thus, LC-MS/MS-based lipid oxidation analyses continue to be actively applied to emulsions and dairy products.

### 5.4. Oleosomes

Oleosomes are lipid storage organelles found in plant seeds. Their cores are composed of TGs and surrounded by a PL monolayer containing proteins such as oleosins [98]. In this review, oleosomes are mainly considered naturally organized food lipid structures for evaluating lipid oxidation and oxidative stability. Isolated oleosomes can also be used as food ingredients or delivery systems for lipophilic compounds [98].

The oxidative stability of oleosomes is influenced not only by the fatty acid composition of their internal TGs but also by phenolic compounds and storage proteins [98,99,100]. In a storage study of rapeseed oleosomes, hydroperoxide formation in TGs occurred more slowly in oleosome O/W emulsions than in bulk oil and lecithin-stabilized O/W emulsion controls, indicating that TGs present within oleosome structures had relatively greater oxidative stability [99]. In sunflower oleosomes, TG oxidation was suppressed under conditions with higher phenolic content, and the rate of lipid oxidation also decreased when storage-protein content was higher [100].

Studies have also evaluated structural changes in oleosomes together with lipid oxidation. During pecan storage, disruption of oleosome structure increased contact between internal lipids and oxygen and thereby promoted oxidation, demonstrating an association between structural stability and oxidative stability of oleosomes [101]. In another study, UPLC-Q Exactive MS-based lipidomics of pecans was used to characterize storage-induced changes in various lipid species, including GLs, PLs, and oxidized phospholipid (OxPL) species, and investigate lipid hydrolysis and oxidation during storage [77].

LC-MS/MS-based lipidomics has also been applied to characterize individual lipid species constituting oleosomes. In rice bran-derived oil bodies, ultra-high-performance liquid chromatography (UHPLC)-Q-Exactive Orbitrap MS-based lipidomics was used to characterize lipid composition, including PLs and GLs, demonstrating that the lipid-species composition of the oleosome interface is closely associated with oxidative stability [102].

Current studies of oleosome oxidation have largely focused on hydroperoxide-based oxidation indices and changes in interfacial structure, whereas direct investigations of individual OxTG and OxPL species in oleosomes remain relatively limited. Future LC-MS/MS-based lipidomics studies may therefore help clarify oleosome oxidation in both native and isolated systems. Representative targeted and untargeted applications across food models are summarized in Table 3 and Table 4, respectively.

## 6. Application of Lipidomics to Lipid Oxidation Analysis in Animal Models 

Lipid oxidation products analyzed in foods are also relevant to biological systems, where related oxidized lipid species can be generated through enzymatic and non-enzymatic oxidation. LC-MS/MS-based lipidomics has therefore been applied to characterize these products in plasma and tissues under oxidative stress and disease conditions [103]. Selected animal studies are included to extend the analytical framework described for food oxidation to biological systems, while considering that the formation and fate of oxidized lipids can also be influenced by enzymatic processes and tissue-specific metabolism.

### 6.1. Oxidative Stress and Disease-Associated Lipid Oxidation

Animal models are useful because oxidative stress can be experimentally controlled and oxidized lipids can be compared across different tissues [103]. In one study, arachidonic acid-derived isoprostane isomers were quantified in plasma by LC-MS/MS after oxidative stress was induced in mice with doxorubicin or tert-butyl hydroperoxide [104]. Isoprostane levels increased under oxidative stress, demonstrating that LC-MS/MS can be used to quantify specific oxidation products in biological samples.

Oxidized lipids generated during low-density lipoprotein (LDL) oxidation have also been analyzed using MS. Because LDL contains diverse lipid classes, including cholesteryl esters (CEs), TGs, and PLs, various oxidized lipids can be generated during oxidation [78]. Minimally oxidized LDL (mmLDL) was analyzed by LC-MS, and oxidized CEs, including cholesteryl ester hydroperoxides (CEOOHs), were identified [79]. A separate study also detected oxidized CEs in atherosclerotic lesions from apolipoprotein E (ApoE)-deficient mice [80].

Disease-associated lipid oxidation has also been investigated in other tissues. In a mouse model of nonalcoholic steatohepatitis, LC-MS-based lipidomics was used to analyze lipids and lipid hydroperoxides in the liver and kidney, identifying disease-associated lipid species including hydroperoxides derived from TGs and PCs [81]. In a rat model of osteoarthritis, oxylipins and endocannabinoids were analyzed by LC-MS/MS in multiple tissues [105]. Tissue-dependent changes were observed, including increases in 12-hydroxyeicosatetraenoic acid (12-HETE) in the knee joint and 15-HETE in the dorsal root ganglia.

Because these studies compare oxidized lipids across different tissues and experimental conditions, pre-analytical handling is also important for reliable interpretation. Oxidized lipids, particularly hydroperoxides, can be unstable during tissue collection and extraction. Rapid cooling or freezing after collection, limited exposure to light and oxygen, low-temperature extraction, and early addition of appropriate internal standards can help minimize changes after sampling [8,25].

### 6.2. Diet-Related Lipid Changes and Dietary Oxidized Lipids

In addition to endogenous and disease-associated lipid oxidation, lipidomics has also been applied to characterize lipid changes induced by diet. In mice with high-fat-diet-induced obesity and insulin resistance, LC-MS/MS-based lipidomics revealed changes in multiple lipid species in cardiac tissue, including TGs [106]. Laser desorption/ionization mass spectrometry (LDI-MS) was also used to determine the spatial distribution of these lipid changes within tissue sections.

However, diet-induced changes in tissue lipid composition should be distinguished from the biological effects of oxidized lipids directly consumed from foods. Food-derived oxidized lipids undergo digestion, absorption, and metabolism and therefore require specific dietary-exposure studies [107]. This distinction is important when relating food lipid oxidation to changes observed in biological systems.

## 7. AI-Assisted Lipidomics Analysis and Applications

LC-MS/MS-based untargeted lipidomics can generate hundreds to thousands of lipid features in a single analysis, making data processing from peak detection and alignment to identification and selection of major lipid species time- and labor-intensive. Here, a feature refers to a single LC-MS signal characterized by a specific *m/z* and retention time. To process these data more rapidly and accurately, AI approaches, including machine learning (ML) and deep learning, have been applied at multiple stages of lipidomics analysis [108]. These applications can be considered according to the analytical workflow, including lipid identification and retention-time prediction, oxidized-lipid profiling and biomarker selection, and model validation and interpretation.

### 7.1. Lipid Identification and Retention-Time Prediction

At the lipid identification stage, ML models have been developed to predict lipid classes by learning fragment-ion patterns generated in MS/MS. A representative example is MS2Lipid, which was developed to classify lipid subclasses using manually curated MS/MS spectra and achieved an accuracy of 97.4% on the test dataset [109]. Unlike conventional approaches that identify lipids based on the presence or absence of specific fragment ions, MS2Lipid learns accumulated MS/MS spectral patterns to predict lipid classes. This approach can facilitate lipid classification when diagnostic fragment ions are unclear or when similar fragments occur across multiple lipid classes.

ML has also been applied to predict lipid retention times in HPLC. Studies have trained ML models using molecular descriptors that numerically represent lipid structural properties, such as molecular weight, carbon number, number of double bonds, and polarity, to predict the retention time of individual lipids [110]. In untargeted lipidomics, multiple lipid candidates may have identical or similar *m*/*z* values. In such cases, comparing retention times predicted from structural properties with experimentally measured values can help narrow candidate lists and improve confidence in lipid identification. However, these predictions should be used as supporting information rather than as substitutes for diagnostic MS/MS or authentic standards. 

### 7.2. Oxidized-Lipid Profiling and Biomarker Selection

More recently, AI-based approaches have also been applied to LC-MS/MS analysis of oxidized lipids. One study developed an LC-MS workflow for OxPL analysis by combining an ML-based retention-time prediction model with the oxidized-lipid identification software LPPtiger 2 [111]. Briefly, lipid retention times were predicted using a model trained on the relationship between PL structure and retention time, and the predictions were then used to construct a candidate OxPL library.

ML can also be used to select major lipid species from oxidized-lipid datasets obtained by LC-MS/MS. In a study of plasma from patients with sepsis-associated acute kidney injury, oxidized lipids were analyzed by UPLC-MS/MS, after which least absolute shrinkage and selection operator (LASSO) and random forest (RF) algorithms were used to select key lipid species associated with patient status from a large number of detected lipids [112]. LASSO and RF are methods for selecting variables that contribute strongly to discrimination among groups, and similar approaches can be used to identify lipid species that best explain oxidative status among lipids detected by LC-MS/MS. However, lipid features selected by these models require further analytical identification and independent validation before they can be used as reliable biomarkers.

### 7.3. Model Validation and Interpretation

The performance of AI-based lipidomics depends strongly on the quantity and quality of the training data, and insufficient or biased datasets can lead to incorrect predictions [113]. Because lipidomics datasets often contain a large number of features relative to the number of samples, overfitting can occur when the model fits the training data but does not perform well on independent data. Small or imbalanced datasets and training data obtained from limited sample types or analytical conditions can also introduce model bias [108,113].

Data leakage is another important problem because information from test samples may unintentionally be used during preprocessing or feature selection, resulting in overestimated model performance [114]. Therefore, preprocessing and feature selection should be separated between training and test datasets. Cross-validation is useful for internal model evaluation, whereas independent test data and external validation are required to more reliably evaluate model performance [114,115].

External validation is also important when models are applied to data obtained from different analytical batches, instruments, or laboratories. Differences in retention time, ionization conditions, MS acquisition, and data processing can reduce model transferability and should therefore be considered when applying a model to new datasets [108,115].

In addition, complex models such as deep learning can suffer from a black-box problem, making it difficult to determine which lipids or MS signals contributed to a prediction [116]. To address this issue, explainable AI methods such as SHapley Additive exPlanations (SHAP), which quantify the contribution of individual features to model predictions, have recently been applied [116]. However, the importance of a feature in an AI model does not confirm its chemical identity or biological significance. Therefore, major lipid features identified by AI should also be evaluated using appropriate lipid annotation and analytical evidence.

**Figure 3:**
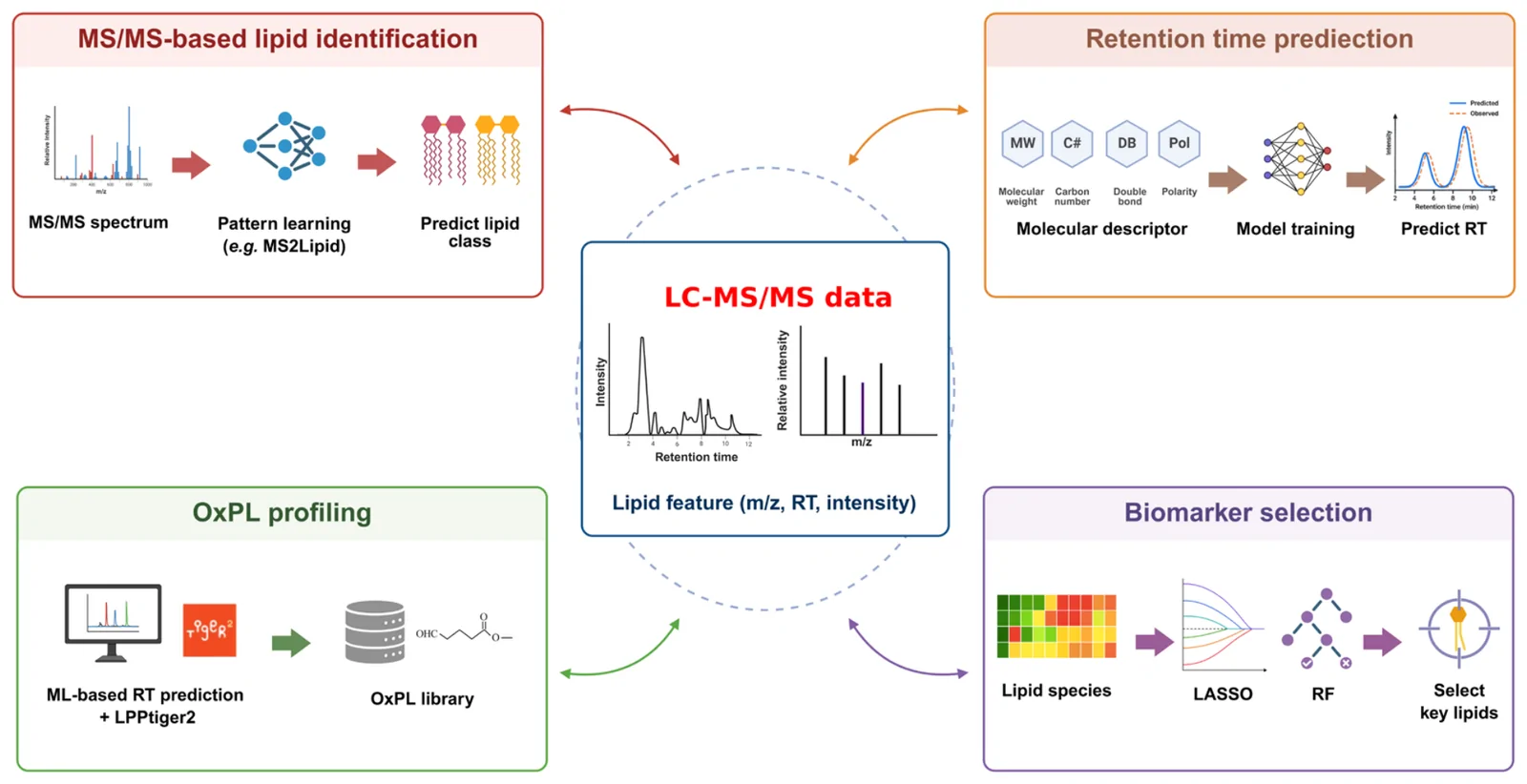
AI-assisted approaches in LC-MS/MS-based lipidomics for oxidized lipid analysis. Representative applications include MS/MS-based lipid class prediction, retention-time prediction, machine-learning-assisted oxidized phospholipid profiling, and biomarker selection from lipidomics datasets. Abbreviations: AI, artificial intelligence; ML, machine learning; RT, retention time; OxPL, oxidized phospholipid; LASSO, least absolute shrinkage and selection operator; RF, random forest.

For reproducibility, studies should clearly report major preprocessing and feature-selection procedures, training and test-set separation, validation strategy, and model parameters [115]. Representative AI-assisted applications across the lipidomics workflow are summarized in Figure 3.

Accordingly, high classification accuracy obtained by internal cross-validation within a single dataset should not be interpreted as evidence of generalizable performance. Data leakage can inflate apparent accuracy, overfitting is favored when the number of lipid features greatly exceeds the number of samples, and instrument- or batch-specific signal patterns may be learned instead of chemically meaningful features. Robust AI-assisted lipidomics therefore requires separation of training and test preprocessing, appropriate nested or independent validation when feature selection is performed, and external evaluation across batches, instruments, laboratories, or sample populations whenever the intended application extends beyond the original dataset [108,113,114,115].

## 8. Conclusions

Lipid oxidation analysis should be performed using complementary analytical approaches selected according to the research objective. Conventional oxidation indices provide information on overall oxidative status, whereas MS-based approaches enable more specific or broader characterization of individual oxidation products and lipid species. No single analytical method can fully characterize the complex progression of lipid oxidation.

Reliable interpretation of lipid oxidation requires consideration not only of instrumental analysis but also of sample handling, matrix effects, analyte stability, and lipid-identification confidence. Hydroperoxides and reactive aldehydes can change during extraction and storage, while differences in food structure and lipid organization can influence oxidation behavior. Therefore, appropriate internal standards, validation of recovery and matrix effects, and structural evidence from MS/MS or authentic standards are important for improving analytical reliability.

Future studies should focus on the molecular characterization of oxidized complex lipids, including OxPLs and OxTGs, as well as epoxy- and oxo-lipids that remain less extensively characterized. Greater standardization of sample preparation, analytical validation, and lipid annotation is also needed to improve comparability among studies. In addition, integration of targeted and untargeted lipidomics with conventional oxidation indices and information on food structure may provide a more comprehensive understanding of lipid oxidation. AI-assisted lipidomics may further support lipid identification and biomarker selection; however, external validation, reproducibility, and transferability across analytical platforms remain important priorities for its broader application.

## Figures and Tables

**Table 1:** Critical comparison of analytical strategies for lipid oxidation studies.

Analytical Method	Target Analyte	Specificity/Sensitivity	Matrix Interference	Quantitative Capacity/Standards	Sample Preparation/Routine Food-QC Suitability	Cost/Throughput
Conventional indices	Global oxidation status	Low–moderate specificity; method-dependent sensitivity	Often high in complex or colored matrices	Global/relative; assay-specific calibration	Simple; limited cleanup; high suitability for routine food-QC screening	Low cost; high throughput
Targeted LC-MS/MS	Defined oxidation products or isomers	Generally high specificity and sensitivity when appropriately validated; analyte- and platform-dependent	Ion suppression; matrix-dependent	Absolute/relative; authentic or isotope-labelled standards preferred	Extraction/cleanup; derivatization if needed; moderate suitability for targeted or confirmatory food-QC	High cost; moderate throughput
Untargeted lipidomics (HRMS)	Broad native and oxidized lipid profile	Moderate specificity; analyte/platform-dependent sensitivity	Ion suppression; matrix-dependent	Mainly relative/semiquantitative; limited standards	Comprehensive lipid extraction; low suitability for routine food-QC	High cost; low throughput
Pseudotargeted lipidomics	Preselected lipid/feature panel	Moderate–high specificity; analyte-dependent sensitivity	Matrix-dependent	Relative/absolute; standards if available	Targeted-like workflow after feature selection; moderate suitability for predefined food-QC marker panels	Moderate–high cost; moderate throughput
Combined strategy	Global indices + defined markers + lipid profile	Broadest coverage; method-dependent	Method- and platform-dependent	Relative + quantitative; multiple standards	Multiple analytical workflows; low suitability for routine food-QC	Highest cost; low overall throughput

Note: POV, peroxide value; TBARS, thiobarbituric acid-reactive substances; p-AV, p-anisidine value; HRMS, high-resolution mass spectrometry; SPME, solid-phase microextraction; QC, quality control.

**Table 2:** Matrix-specific characteristics and applications of lipidomics in food lipid oxidation studies.

Food Matrix	Major Lipid Characteristics	Lipidomic Changes	Application
Edible oils	Mainly TGs; different fatty-acyl compositions	Changes in PUFA-containing TGs and oxidized lipid species	Storage and processing
Meat	TGs and PLs	Changes in PUFA-containing lipid species	Processing and storage
Dairy products	TGs and PLs	Changes in individual TG and PL species	Processing and storage
Emulsions	Dispersed lipids; interfacial organization	Changes in lipid species according to physical organization	Oxidation stability
Oleosomes	TGs and PLs; organized lipid structure	Changes in TG and PL species	Oxidation stability

Note: TG, triacylglycerol; PL, phospholipid; PUFA, polyunsaturated fatty acid.

**Table 3:** Representative targeted LC-MS/MS analyses of lipid oxidation in different food models.

Food Matrix	Specific Sample	Extraction Method	Analytical Instrumentation	Target Analyte	Ref.
Edible oil	Soybean, rice bran, and olive oil	Lipase AY-30 hydrolysis followed by recovery of the free fatty acid fraction	LC-micrOTOF-Q II Q-TOF MS (Bruker), CHIRALPAK IB N-3 column (3 µm, 2.1 × 150 mm), ESI, SRM, post-column sodium acetate addition to form [M + Na]^+^	Linoleic acid hydroperoxide (HpODE) isomers	[7]
Edible oil	Canola oil	Hexane dilution followed by direct injection	LC-QTRAP, Inertsil SIL-100A, ESI, MRM, post-column sodium acetate addition	TGOOH isomers	[19]
Edible oil	Olive oil	Hexane dilution followed by direct injection	LC-QTRAP-MS/MS, Inertsil SIL-100A column (5 µm, 2.1 × 250 mm), ESI, MRM, post-column sodium acetate addition	TGOOH isomers	[73]
Edible oil	10 commercial fish oil supplements	2,4-DNPH derivatization followed by SPE cleanup	LC-MS/MS (triple quadrupole), reversed-phase C18 column, targeted analysis	Aldehyde and ketone derivatives	[24]
Meat	Pork and processed pork products	Homogenization followed by SPE cleanup	HPLC-MS/MS, reversed-phase liquid chromatography column	4-HNE	[10]
Meat	Precooked Chinese stewed beef	Organic-solvent-based total lipid extraction	UHPLC-ESI-MS/MS, charged surface hybrid (CSH) C18 column (1.7 µm, 2.1 × 100 mm)	GL/PL precursors and oxidation-related lipid species associated with warmed-over flavor (WOF)	[94]
Emulsion (infant formula)	Canola-oil-based infant formula and canola oil + DHA ethyl ester-fortified infant formula	Methanol extraction followed by SPE cleanup	UPLC-MS/MS, reversed-phase UPLC column, negative ESI, MRM	Total, free oxylipins	[96]
Emulsion (infant formula)	Refrigerated storage samples of canola-oil-based and DHA-fortified infant formula	Methanol extraction of free oxylipins with SPE cleanup; the esterified fraction was hydrolyzed and assessed as total oxylipins	UHPLC-triple quadrupole (QqQ)-MS/MS, C18 column (1.8 µm, 2.1 × 150 mm), ESI	Esterified oxylipins	[97]
Emulsion (mayonnaise)	3 commercial mayonnaise products	CHCl3/MeOH/H2O (8:4:3, v/v/v) extraction followed by aminopropyl SPE fractionation	LC-4000 QTRAP MS/MS (SCIEX), Atlantis T3 column (3 µm, 2.1 × 100 mm), ESI, MRM	PC hydroperoxide (PCOOH; 16:0/13-HpODE-PC, 16:0/9-HpODE-PC)	[74]
Emulsion (milk)	Commercial milk	Organic solvent lipid extraction	LC- MS/MS (SCIEX), 5C18-MS-II column (5 µm, 2.0 × 250 mm), ESI, MRM	TGOOH isomers	[75]

Note: 2,4-DNPH, 2,4-dinitrophenylhydrazine; DHA, docosahexaenoic acid; ESI, electrospray ionization; HpODE, hydroperoxyoctadecadienoic acid; SRM, selected reaction monitoring; MRM, multiple reaction monitoring; PCOOH, phosphatidylcholine hydroperoxide; Q-TOF, quadrupole time-of-flight; SPE, solid-phase extraction; TGOOH, triacylglycerol hydroperoxide; UPLC, ultra-performance liquid chromatography.

**Table 4:** Representative untargeted lipidomics-based analyses of lipid oxidation in different food models.

Food Matrix	Specific Sample	Extraction Method	Analytical Instrumentation	Target Analyte	Ref.
Edible oil	Flaxseed oil	n-Hexane, ultrasound extraction	UPLC-Q Exactive Orbitrap MS, Cortecs C18 column	GL, PL, TG and oxidation-related lipidomic profile	[91]
Edible oil	Hazelnut oil	MTBE	UPLC-QTOF-MS, reversed-phase C18 column, untargeted lipidomics	TG, DG, PC, and PE species	[92]
Meat	Yellow-feathered chicken	Organic-solvent-based total lipid extraction	UPLC-ESI-MS, reversed-phase UPLC column, untargeted lipidomics	TG, DG, PC, PE, LPC, and lysophosphatidylethanolamine (LPE) species responsive to thermal processing	[93]
Oleosome	Pecan kernels	Organic solvent lipid extraction	UPLC-Q Exactive MS, CSH C18	GL, PL, OxPL, fatty acid ester of hydroxy fatty acid (FAHFA), and other lipid species changing during storage	[77]
Oleosome	Rice bran oil bodies	Folch/Bligh–Dyer lipid extraction	UHPLC-QTOF-MS/MS, reversed-phase UHPLC column	Lipidomic signatures including PL and GL	[102]
Dairy (cheese)	Camembert	Folch lipid extraction	UHPLC-Q-Orbitrap-MS, Accucore C30 column (2.6 µm, 2.1 × 150 mm)	Lipidomic profile including OxTG and OxDG	[76]

Note: DG, diacylglycerol; FAHFA, fatty acid ester of hydroxy fatty acid; GL, glycerolipid; HRMS, high-resolution mass spectrometry; LPC, lysophosphatidylcholine; LPE, lysophosphatidylethanolamine; OxDG, oxidized diacylglycerol; OxPL, oxidized phospholipid; OxTG, oxidized triacylglycerol; PC, phosphatidylcholine; PE, phosphatidylethanolamine; PL, phospholipid; MTBE, methyl tert-butyl ether extraction; QTOF, quadrupole time-of-flight; TG, triacylglycerol; UPLC, ultra-performance liquid chromatography.

## Data Availability

No new data were created or analyzed in this study. Data sharing is not applicable to this article.

## Abbreviations

The following abbreviations are used in this manuscript:

2,4-DNPH	2,4-dinitrophenylhydrazine
4-HHE	4-hydroxy-2-hexenal
4-HNE	4-hydroxy-2-nonenal
AI	artificial intelligence
APCI	atmospheric pressure chemical ionization
ApoE	apolipoprotein E
APPI	atmospheric pressure photoionization
CE	cholesteryl ester
CEOOH	cholesteryl ester hydroperoxide
CSH	charged surface hybrid
CV	coefficient of variation
DG	diacylglycerol
DHA	docosahexaenoic acid
ESI	electrospray ionization
FA	fatty acid
FAHFA	fatty acid ester of hydroxy fatty acid
FAOOH	fatty acid hydroperoxide
GC	gas chromatography
GC-MS	gas chromatography-mass spectrometry
GL	glycerolipid
HDoHE	hydroxydocosahexaenoic acid
HETE	hydroxyeicosatetraenoic acid
HILIC	hydrophilic interaction liquid chromatography
HODE	hydroxyoctadecadienoic acid
HpDoHE	hydroperoxydocosahexaenoic acid
HPLC	high-performance liquid chromatography
HPLC-MS/MS	high-performance liquid chromatography-tandem mass spectrometry
HpODE	hydroperoxyoctadecadienoic acid
HRMS	high-resolution mass spectrometry
KEGG	Kyoto Encyclopedia of Genes and Genomes
LASSO	least absolute shrinkage and selection operator
LC-MS	liquid chromatography-mass spectrometry
LC-MS/MS	liquid chromatography-tandem mass spectrometry
LDI-MS	laser desorption/ionization mass spectrometry
LDL	low-density lipoprotein
LOD	limit of detection
LOH	hydroxy fatty acid
LOOH	lipid hydroperoxide
LOQ	limit of quantification
LOX	lipoxygenase
LPC	lysophosphatidylcholine
LPE	lysophosphatidylethanolamine
MDA	malondialdehyde
ML	machine learning
mmLDL	minimally oxidized low-density lipoprotein
MRM	multiple reaction monitoring
MS	mass spectrometry
MS/MS	tandem mass spectrometry
MTBE	methyl tert-butyl ether
O/W	oil-in-water
OPLS-DA	orthogonal projections to latent structures discriminant analysis
OxDG	oxidized diacylglycerol
OxPL	oxidized phospholipid
OxTG	oxidized triacylglycerol
p-AV	p-anisidine value
PAH	polycyclic aromatic hydrocarbon
PC	phosphatidylcholine
PCA	principal component analysis
PCOOH	phosphatidylcholine hydroperoxide
PE	phosphatidylethanolamine
PL	phospholipid
PLS-DA	partial least squares discriminant analysis
POV	peroxide value
PUFA	polyunsaturated fatty acid
QC	quality control
QqQ	triple quadrupole
QTOF	quadrupole time-of-flight
RF	random forest
RSD	relative standard deviation
RT	retention time
SHAP	SHapley Additive exPlanations
SPE	solid-phase extraction
SPME	solid-phase microextraction
SRM	selected reaction monitoring
TBARS	thiobarbituric acid reactive substances
TG	triacylglycerol
TGOOH	triacylglycerol hydroperoxide
TLC	thin-layer chromatography
UHPLC	ultra-high-performance liquid chromatography
UHPLC-MS/MS	ultra-high-performance liquid chromatography-tandem mass spectrometry
UPLC	ultra-performance liquid chromatography
VIP	variable importance in projection
W/O	water-in-oil
WOF	warmed-over flavor
